# Unconditional Cash and Family Investments in Infants: Evidence from a Large-Scale Cash Transfer Experiment in the U.S.

**DOI:** 10.21203/rs.3.rs-2507540/v1

**Published:** 2023-02-07

**Authors:** Lisa A. Gennetian, Greg Duncan, Nathan A. Fox, Sarah Halpern-Meekin, Katherine Magnuson, Kimberly G. Noble, Hirokazu Yoshikawa

**Affiliations:** Duke University, Sanford School of Public Policy, 201 Science Drive, Durham, NC 27708; University of California, Irvine, School of Education, 401 E. Peltason Drive, Suite 3200, Irvine, CA 92617; University of Maryland, College Park, College of Education, 3119 Benjamin Building, College Park, MD 20742; University of Wisconsin-Madison, Human Development and Family Studies, 4107 Nancy Nicholas Hall, Madison, WI 53706; University of Wisconsin-Madison, Sandra Rosenbaum School of Social Work, 1350 University Ave., Madison, WI 53706; Columbia University, Teachers College, 525 West 120th Street, New York, NY 10027; New York University, Steinhardt School of Culture, Education, and Human Development, 82 Washington Square E, New York, NY 10003

**Keywords:** D13, H31, I30, J13, J18, J22, policy, poverty, randomized controlled study, children, family investment, household production and intrahousehold allocation

## Abstract

Economists have limited causal evidence on how families receiving unconditional income would spend those funds. We examine financial and time investments in infants among families living in poverty from a large-scale, multi-site randomized controlled study of monthly unconditional cash. We find increased spending on child-specific goods and mothers’ early-learning activities with their infants. The marginal propensity to consume child-focused items from the cash transfer exceeded that from other income, consistent with the behavioral cues in the design. We find no statistically detectable offsets in household earnings or impacts on pre-registered outcomes related to expenditures, labor supply, childcare or subjective well-being.

How people residing in poverty spend their money and time has long animated public and political debate in the U.S. Economists wrestle with competing goals of designing policies that will reduce the consequences and social costs of poverty yet will not reinforce or fuel harmful behaviors or long-term dependence on government aid (National Academies of Sciences, Engineering, and Medicine 2019; Evans and Popova 2017; Hammond and Xu 2021; Winship 2021). While economists have demonstrated how child-directed services and parenting-skill programs produce economically significant returns (e.g., Algan et al., 2022; Conti, Heckman and Rodrigo, 2016), much less is understood about how direct cash support affects family investments in goods and time. Causal evidence on how people spend additional income upon receipt of government support is limited even as the question of how money is spent has elevated in importance as U.S. policy makers consider embarking on strategies to expand income support beyond historical in-kind benefits. This study expands and extends crucial insights from the U.S. negative income tax experiments of the 1960s through the 1980s (Levine et al., 2005) by uniquely examining unanswered questions about family time and money responses to direct income support in a contemporary diverse target population.

Identifying causal impacts of unconditional cash transfers on family spending and time allocations in the U.S. is complicated by the fact that the largest safety-net and income support policies are conditioned on behavior such as employment. As a result, uncoupling the effect of net income from the effect of other behaviors such as earning money or indirect benefits from consuming more or higher-quality goods is methodologically difficult.^1^ Second, other types of studies that aim to identify key mechanisms related to investments in children are not well suited to assess overall impacts on time and money investments in response to income either because they examine specific parental behaviors or parenting skills, such as taking a child to a doctor’s appointment, ensuring attendance at school, or interacting directly with a child (Del Boca, Flinn, and Wiswall 2014; Francesconi and Heckman 2016); and, even in some cases of randomized control studies, challenges remain for causal inference (e.g. Conti, Heckman and Rodrigo, 2021).

The present study identifies the causal impact of monthly unconditional cash transfers on time and money investments via a large-scale multi-site randomized controlled trial (RCT) that launched in 2018. Specifically, 1,000 racially and ethnically diverse mothers in four urban metropolitan areas in the United States were recruited in postpartum wards of hospitals shortly after giving birth. After consenting to participate in the research study and to receive a cash gift, they were randomized to receive a monthly unconditional cash transfer of either $333 or $20 for the first several years of their child’s life.^2^ Over $6 million dollars have been successfully distributed on a monthly basis to families in this RCT so far. The high cash gift is financially significant, amounting to a 19 percent increase in income at the federal poverty level. The experiment was also designed to minimize the impact of the cash transfer on eligibility for other government support (which required passage of new legislation in some states).

Marshalling insights from behavioral economics, the cash transfer was designed to be sustained, foreseeable, automatic, and predictable. Unlike the administrative burdens present in many U.S. anti-poverty programs, documentation and eligibility requirements were minimal with no subsequent re-evaluation of circumstances for recertification over the course of cash transfer receipt. As described later, uptake of the cash transfer among families in the study is universal, including many families who otherwise are ineligible for government benefits (e.g., because of documentation or earnings requirements). While the cash transfers were unconditional, the disbursement cycle and branding of the debit card incorporated child-specific behavioral cues.

This paper specifically reports on impacts on family time and money investments during the first year after birth. This time period is of particular economic significance: family income sharply drops after childbirth (Stanczyk 2020), parent allocation of time in the labor force and child rearing faces the most demands, child-related expenses are highest, and the return on private and public investment in children’s early development is especially high (Heckman and Mosso 2014; Heckman 2007).

Results demonstrate that families receiving the unconditional high cash gift increased the amounts of money and time spent on and with infants. Specifically, expenditures on child-focused items such as books, toys, diapers, and children’s clothing were larger in high-cash-gift families than in low-cash-gift families, and for these items, the marginal propensity to consume the cash gift income was larger than the marginal propensity to consume on those items from other sources of income. In open-ended semi-structured interviews, mothers described the cash gifts as distinct from other sources of government income and explicitly discussed mental earmarking of this money for the baby. Moreover, more mothers spent time in early learning activities (e.g., reading books, telling stories and play) with their infant with no detectable shift in maternal time in paid work or infant’s time in nonparental childcare. The impact on time spent in early learning activities is equivalent to a 10% increase in minutes per week, and, as such, reduces by 15% the gap between low versus highly educated mothers in time spent with children on teaching activities associated with children’s language development (Kalil et al. 2012).

Household income was higher in high- versus low-cash-gift families, decreasing the proportion of families residing 100 percent or below poverty, indicating little substantive offset from reductions in household earnings or income from government assistance programs. Nevertheless, mean income remained below 200% of the federal poverty threshold for the majority of these families with young children. With the exception of child-focused expenditures, propensity to spend on other pre-registered outcomes, including on alcohol or cigarettes, did not differ between these groups at conventional levels of statistical significance. Aspects of economic hardship such as homelessness, evictions, and missed bill payments did not statistically differ between high- and low-cash-gift families. High-cash-gift mothers did not report less worry over finances or improved subjective well-being in terms of happiness or optimism. Although mothers talked about the “miracle” of the cash gift, they also talked about financial strain as top of mind.

The results provide two distinct contributions regarding how money and time are spent in response to cash support. First, the findings offer empirical evidence of how predictable monthly unconditional cash impacts spending in ways that are positively directed toward children in a contemporary U.S. context, thus complementing similar evidence generated from quasi-experimental studies such as those of maternal pensions (Duflo 2003; Aizer et al. 2016) and income transfers to Indigenous populations (Akee et al. 2010). The child-focused design of the cash gift in this study may have particularly encouraged complementarities in household investments in child-specific goods and time spent with child. Another possibility is that the behavioral cues in the child-focused design coupled with the high cash gift made salient a parenting identity that translated into such child-specific investments (Akerlof and Kranton 2010). Notably, these investments are occurring during the economically significant first year of children’s development (Knudsen et al. 2006; Heckman 2007). Second, the findings reveal psychological, or non-financially binding, ways that people may treat money in their spending allocations that is counter to conventional economic assumptions about the fungibility of cash irrespective of its design. The unconditional high cash gift in this study was directed toward a variety of purchases but most substantively toward a range of child-specific expenditures.

## I. Background

By generating causal evidence, this study complements predictions and prior research on the economic model of family investment (e.g., Becker and Tomes 1976; Cunha and Heckman 2007; Del Boca, Flinn, and Wiswall 2014; Francesconi and Heckman 2016; Heckman and Mosso 2014; Doepke, Sorrenti, and Zilibotti 2019; Caucutt and Lochner 2021). As indicated in a conventional family investment model, the proportion of the transfer consumed or invested in children will depend on household preferences, total household resources, and the productivity of investments. Unconditional income as examined in this study has three features that complicate predictions from existing conventional economic models.

First, preferences across or within families may be heterogeneous, and how opportunities to act upon preferences in response to unrestricted cash income might differ by context and family circumstances (Attanasio and Kaufmann 2009; Banerjee and Duflo 2019; Mullins 2019). For example, spending or investing increased financial (liquid) resources might be constrained by historical and current structural and systemic barriers that limit choices and options. Food deserts and housing segregation marked by historical redlining serve as examples in the U.S. (e.g., Karpyn et al. 2019). Further, household composition can affect allocations toward any one member, such that a sibling or a resident adult may draw resources away from investment in a specific child or may alleviate economic burden and thus free up resources for child investments (Aizer and Cunha 2012; Altmejd et al. 2021). Finally, household members’ control over resources and resource allocation decisions can shape how resources are spent, as shown in studies of women’s intra-household bargaining power associated with increased spending on goods and services that benefit children (Attanasio and Lechene 2002; Lundberg 2008; Browning, Chiappori, and Weiss 2014).

Second, cognitive resources and the mental drain of financial scarcity are likely to affect family resource allocation decisions and preferences around money and time use, particularly when financial scarcity is coupled with the mental drain of parenting (Mullainathan and Shafir 2013; Haushofer and Fehr 2014; Gennetian and Shafir 2015).^3^ This framework posits that parents’ preferences that feed into allocation decisions and affect behavior are complicated by intentions and actions influenced by broader social norms and expectations, along with decision-making inertia, myopia, and calibrations of risk.^4^ These propositions, informed by behavioral economics, further raise the specter of mental accounting; that is, the notion that people may earmark and spend money differently based on its origin, or based on social signals with respect to its intended use (Akerlof and Kranton 2010; Romich and Weisner 2000; Zelizer 2011). These insights complicate predictions from conventional economic models with respect to unconditional income, including the extent to which children benefit from unconditional income identified as a “child benefit.”

Third, how cash transfers are received is not psychologically or socially neutral. Lump-sum transfers may be spent differently than monthly disbursements of income, and, as previously mentioned, labeling can invoke certain social signals, norms, or expectations on how resources are to be used. Thus, unrestricted income may not be as fungible as presumed by standard microeconomic demand theory. For example, the Dutch child benefit is associated with a higher propensity to consume on children’s clothing relative to other income sources, a pattern not shown for items such as adult clothing (Kooreman 2000). A cash transfer labeled as “education support” given to fathers of school-aged children in poor rural communities in Morocco produced large gains in school participation, posited to be due to a shift in parental beliefs in educational investment; in contrast, a similarly labeled conditional cash transfer targeting mothers had no statistically detectable impact (Benhassine et al. 2015). Patterns of parental expenditures can be complex: analyses of the lump-sum EITC demonstrate substantial family heterogeneity in spending, with some prioritizing child-specific goods (e.g., children’s clothing, private school tuition) or establishing a savings account for their child and others allocating the EITC refund to general household consumption and debt repayment (Romich and Weisner 2000).

## II. The Baby’s First Years randomized controlled trial

Baby’s First Years (BFY) is a large-scale multi-site longitudinal unconditional cash transfer randomized controlled trial, the first of its kind in the U.S. Mothers in the treatment group (termed the “high-cash-gift group”) receive monthly gifts of $333 ($3,996/year), while mothers in the low-cash-gift group receive a $20 monthly gift ($220/year). The treatment amount is equivalent to increasing the annual income of a family of three residing at the poverty line ($21,330 in 2019) by approximately 19%. The annual cash gift is similar in magnitude (in today’s dollars) to income supplements experienced by families in prior welfare-to-work experiments, which produced improvements of 0.15 to 0.20 standard deviations on the achievement of preschool to school-aged children (Duncan, Morris, and Rodrigues 2011) and to the average $3,200 lump-sum income transfers to families with children from the EITC, shown to have similarly sized impacts on children’s cognitive outcomes (Dahl and Lochner 2012). As feasible, agreements were secured with state and local officials to minimize risk of the cash gift interfering with eligibility for public benefits, including Temporary Assistance for Needy Families (TANF), Supplemental Nutrition Assistance Program (SNAP), Medicaid, childcare subsidies, and Head Start. In two of the four sites, we secured state legislation to ensure this; other sites relied on administrative strategies in collaboration with the study investigators. Mothers were informed of any risk to their income eligibility for other programs prior to consenting to receive the cash gift.^5^

Upon consent to enroll in the research study, mothers were told about the opportunity to participate in the cash transfer intervention. They were handed a personalized activated Mastercard debit card co-branded with a “4MyBaby” logo. The monthly cash transfer is automatically loaded on the debit card, along with a text reminder, each month on the day of the child’s birth date. Mothers continue to receive the cash transfer on an opt-out (vs. opt-in) basis. The cash transfer is predictable and monthly, thus reducing the mentally taxing nature of income uncertainty and instability prevalent in U.S. low-income households, whether due to the characteristics of low-wage work, the eligibility and recertification requirements of public benefits (Gennetian and Shafir 2015; Hill et al. 2013; Morduch and Schneider 2017), or uncertainty in the amount or timing of benefit receipt.

## III. Sample and data

Participating mothers and infants were recruited from 12 hospitals in four metropolitan areas: New York City, New Orleans, the greater Omaha metropolitan area, and the Twin Cities (Minneapolis and St. Paul). Selection of these metropolitan areas was guided by an aim to enroll a racially and ethnically diverse sample of low-income mothers across geographic regions that vary in cost of living and generosity of state safety-net programs. Eligibility criteria for the study included (1) mother 18 years or older with the exception of Nebraska, where the age of consent was 19 years or older; (2) self-reported household income below the federal poverty threshold in the calendar year prior to the interview, counting the newborn; (3) healthy full-term birth (i.e., 37 weeks’ gestation or greater; not in the NICU; no known developmental or neurological problems); (4) anticipated residence within 50 miles of hospital; (5) social security number or taxpayer ID number for purposes of payment; and (6) proficiency in English or Spanish for the purposes of available child outcome measurement.

A total of 13,483 mothers were identified, 8,243 of whom agreed to be assessed for eligibility through a brief screener (see baseline CONSORT diagram in Appendix Figure A1). Of these, 6,839 did not meet the inclusion criteria, and 341 declined to consent. A baseline interview was completed with the remaining 1,051 mothers. Of these 1,051 mothers, 1,003 agreed to receive cash gifts and were randomized into the high-cash-gift or low-cash-gift groups. Randomization into the high-cash-gift or low-cash-gift group occurred at the site level. Of the 1,003 mothers who were randomized, three were excluded because they notified the interviewer within two days after completing the baseline interview that they wanted to withdraw and stop receiving cash gifts. The result is a final sample of 1,000 mothers and infants recruited between May 2018 and June 2019.

Striking a balance between statistical power and project costs, 40% of the recruited sample within each site was randomized to receive $333 monthly cash gifts and 60% to receive $20 monthly gifts. With an enrolled sample of n=1,000 mother-infant dyads, and accounting for a predicted 20% attrition over longer-term follow-ups, the anticipated sample size of 800 dyads during subsequent waves of data collection is estimated to provide 80% power to detect a .207 standard deviation impact at p<.05 in a two-tailed test on cognitive functioning and family process outcomes.^6^ Randomization successfully achieved baseline equivalence across 30 characteristics for the full enrolled sample of 1,000 mother-infant dyads and within each site (see Appendix Table A1 for full sample; also available in Noble et al. 2021).

The child age 1 survey (hereafter referred to as Wave 1 follow-up) began in July 2019 and continued through June 2020 with an overall 94% completion rate (see Appendix Table A2). Of the 37 characteristics measured at study entry among the Wave 1 sample, four had small statistically significant differences by cash gift group including race/ethnicity (whether identified as Black or American Indian/Eskimo/Aleut), single parent (whether never married, single living with partner, or biological father living in household), alcoholic drinking during pregnancy, and reports of household receipt of benefits (see Appendix Table A3).^7^ Some of these statistical differences have very little substantive meaning by way of magnitude of difference (e.g., household receipt of benefits is 95% versus 97%); nevertheless, as described below, all estimates are adjusted by these baseline characteristics.

The Wave 1 follow-up survey asked mothers about sources of household income and material hardship (capturing health, food, bill paying, housing, consumer durables, and fear of crime [Heflin, Sandberg, and Rafail 2009; Heflin 2017; Iceland, Kovach, and Creamer 2021]) and related household food, utility, and transportation expenditures. These budget items collectively represent the largest share of a typical monthly budget among households with children residing at or below 200% of the federal poverty level (see estimates, for example, in Gennetian, Conwell, and Daniels 2021). Two components of the Wave 1 follow-up captured direct child investments: maternal reports of expenditures on child-specific goods and maternal reports of frequency of time spent with the child on human-capital building activities such as reading, telling stories, and playing. Mothers’ time spent in market work is based on self-reports of labor force participation.^8^

As context for this study’s analysis, the Wave 1 sample (n=931, as shown in Appendix Table A3) is racially and ethnically diverse: per mothers’ self-reports, 39% identify as Black, 42% Latina, <1% Asian/Pacific Islander, 1.5% Native American, 11% white, non-Hispanic, and 7% multiple races/other. Approximately one out of three infants were first-time births for the mother, and one out of five mothers reported being married. Nearly 60% of mothers worked for pay while pregnant, and 92% reported plans to return to work. Forty-one percent reported that the biological father of the infant resided in the household. The average household income, at $22,313, is just above the federal poverty line for a family of three ($21,330 in 2019); thus the cash transfers represent an 18% annual income boost. Less than 13% of mothers reported receiving government cash assistance from the TANF program, whereas over 95% reported receiving some type of government benefit (SNAP, WIC [Special Supplemental Nutrition Program for Women, Infants, and Children], Head Start, other free childcare, Medicaid, housing assistance, unemployment benefits, or other). Eighty percent of families were net worth poor (defined as net worth less than one-fourth of the federal poverty line or having assets sufficient to meet basic needs for three months, as defined by the poverty line; Gibson-Davis, Keister, and Gennetian 2021).

State-level child poverty rates and racial/ethnic populations vary by study sites, and, accordingly, some characteristics of the families show similar variation. Nearly 80% of mothers in the New Orleans sample identify as Black, whereas 87% of mothers in the New York City sample identify as Latina. Mothers who report being married account for 32% in New York City, 24% in the Twin Cities and Omaha sites, and 7% in New Orleans. On the other hand, rates of residence with the baby’s biological father at study entry are substantively similar across sites, as is overall household income and reports of household receipt of government benefits. Therefore, all estimates described later also include a site-specific indicator in addition to individual and family baseline characteristics.

## IV. The cash gift and debit card transactions

The debit card containing the cash gift can be used at ATMs or for any point-of-sale transaction in person or online (Rojas et al. 2020); money from other sources cannot be loaded on the card. We sought consent from mothers to access their transaction data and obtained consent from 839 of the 931 mothers in the Wave 1 sample. It is made clear to mothers that the monthly cash gift will continue regardless of their participation in the research. Indeed, 4MyBaby card activity is observed for four mothers with deceased infants, one of four incarcerated mothers, and all three low-cash-gift mothers who declined to participate in the Wave 1 survey. As of June 2020 (the end of Wave 1 data collection), we have data on over 62,800 transactions.^9^ As shown in Appendix Table A4, over the first 12 months, 3% (n=15) of the low-cash-gift mothers and <1% (n=1) of the high-cash-gift mothers never used the card (from the time of study enrollment or card activation).^10^ In any given month, over 90% of the mothers in the high-cash-gift group used the card. Two-thirds of high-cash-gift mothers used the card every month. Very few transactions failed due to insufficient funds or PIN problems.^11^ Further, most of the cash gift tends to be expended within days of the disbursement, and nearly all of it tends to be spent or withdrawn from the card before the next monthly disbursement. The most common transaction among high-cash-gift recipients is withdrawal of cash from an ATM, averaging $1,435 in withdrawals, representing approximately one-third of the $4,000 received annually. In contrast, $11 of the $220 received annually is withdrawn as cash from an ATM among low-cash-gift recipients. The remainder of the high cash gift is dispersed across a variety of point-of-sale venues, with the largest amounts and most frequent transactions occurring at large chain stores and various food- or grocery-related venues (see Appendix Table A5). One implication of the high amount of ATM cash withdrawal among high-cash-gift recipients is the limited ability to interpret expenditures from the debit card transaction data alone, hence the importance of having additional information from survey data.

## V. Intent-to-treat estimates

The intent-to-treat (ITT) estimates are derived by the following model implicated by the random assignment design:

(1) Y=Zπ+Xβ+ε

where *Z* is an indicator of whether a mother is in the high-cash-gift group, and thus *π* is the causal estimate of receipt of the high-cash gift. *Y* is the outcome of interest for mother or the household and *ε* is the error term. The model includes the following baseline covariates (*X*) with the goal of improving precision in the estimate: mother’s characteristics (mother’s age, maximum education level attained, race and ethnicity, marital status, general health, an indicator of maternal depressive symptoms, and cigarette and alcohol consumption during pregnancy), household characteristics (number of children born to mother, number of adults in the household, father living with the mother, household income, and household net worth), baby’s birth characteristics (weight at birth and gestational age), a site-based fixed effect, and an indicator for the switch from in-person to phone interviews. Robust standard errors are produced via Huber-White adjustments for heteroskedasticity. Given the implementation success of the debit card mechanism, the ITT estimate captures the effects of a net positive income shock of $313, essentially equivalent to a treatment-on-the-treated interpretation.

All main statistical analyses are pre-registered. We address the possibility of false positives by estimating the statistical significance of conceptually similar outcomes; that is, we capture a common or similar domain or “family,” by generating a familywise error rate of outcomes using step-down resampling methods (Westfall and Young 1993; results shown for outcomes in Tables 4 and 5, and in Appendix Table A10). The regression-adjusted model also includes an indicator for whether the Wave 1 follow-up survey was conducted in person or by phone (detailed further below), as well as the child’s age in months at the time of the Wave 1 survey. Child age at the Wave 1 follow-up serves as a within-treatment group proxy for the length of time receiving the cash gift, in case the length of time of cash gift receipt might affect behavior and as a control for child-specific and related outcomes that might differ by child age (e.g., transitioning from breastmilk or formula to milk). While over 60% of the Wave 1 interviews were completed within the intended two-week window before or after the child’s first birthday (equivalent to child age 11 to 13 months), just under 40% occurred when the child was older than 14 months. The average child age at the Wave 1 data collection time was 13.1 months (sd=2.1) for the low-cash-gift group and 12.6 months (sd=1.5) for the high-cash-gift group.

Two potential sample selection considerations with respect to the ITT estimates are related to the Wave 1 data collection. First are the typical considerations with respect to sample attrition and how the 931 mothers that responded to the Wave 1 survey might differ from the 1,000 initially enrolled. Consistent with the high response rate, few statistical differences emerge when the baseline characteristics of the fully enrolled sample are compared with the Wave 1 survey sample both overall and within each site.^12^ The second consideration is related to the COVID-19 pandemic, which had implications for the mode of data collection and the demographic composition of the accumulated sample before versus during the pandemic. By March 16, 2020, when the pandemic forced stay-at-home orders and related disruptions and the Wave 1 follow-up quickly transitioned from in-person to phone interviews, 65.3% of the high-cash-gift group and 56.6% of the low-cash-gift group had completed the Wave 1 survey in person. Thus, the pandemic generated two potentially confounding complexities for the Wave 1 data: a data collection change from in-person to phone interviews, and a differential experience of being interviewed before the pandemic (in person) vs. after the onset of the pandemic (by phone).

Based on our review of announcements and mobile phone activity, the four metropolitan areas in this study over the March to June 2020 period experienced similar timing of local guidance and stay-at-home ordinances, thus limiting variation in timing across sites that could be captured through more extensive site-by-week interactions. The dichotomous variable (for timing before versus after March 16, 2020) in all regressions should capture any potential residual combined effect of the change in data collection mode due to the pandemic. The transition from in-person to phone interviewing occurred within three days, such that there was very little lag in the pace of data collection. Although some sensitive survey data (e.g., use of substances such as opioids and experiences of domestic abuse) that had been collected via confidential computer-assisted mode could not be quickly converted to phone-based administration, the outcomes that are the focus of this study, all of which have established validity through phone as well as in-person administration, had no disruption. With respect to the second concern, differences in characteristics within each treatment group before and after the onset of the pandemic might matter if the pandemic shifted the experience of receiving the cash gift and the reported outcomes in ways that are systematically correlated with the sample characteristics. There were no statistically discernible discontinuities in the types of transactions observed on the debit cards 30 and up to 60 days prior to and following March 16, 2020, with the exception of an increase in purchases occurring online after the onset of the pandemic as compared with the pre-pandemic period. Baseline characteristics of the pre-pandemic Wave 1 sample are statistically similar to the pandemic-era Wave 1 sample, except that there are more married mothers in the pandemic-era Wave 1 sample. The pre-pandemic Wave 1 sample and the pandemic-era Wave 1 sample also each meet the criteria for baseline equivalence by treatment status.

Nevertheless, we investigated how sensitive the Wave 1 ITT estimates derived from the main specification findings described below may be to these issues by re-estimating the ITT estimates adjusting the main specification with weighting. We applied inverse probability weights to adjust the baseline characteristics of the low-cash-gift group to the baseline characteristics of the high-cash-gift for the n=931 Wave 1 sample, the n=605 pre-pandemic Wave 1 sample, and the n=325 Wave 1 pandemic sample (Appendix Tables A6b–A6d). We applied non-response weight to adjust the n=606 pre-pandemic sample and the n=931 Wave 1 sample to reflect the baseline characteristics of the n=1,000 full study sample (Appendix Tables A6e–A6f). The findings on key selected pre-registered outcomes do not differ in economic or statistical significance with the exception of the within pre-pandemic sample analysis (Appendix Table A6d) signifying the importance of relying on the full Wave 1 (n=931) sample.

## VI. Impacts on household income and family investment

Tables 1 through 5 present the ITT findings on pre-registered outcomes and pre-registered summary indices of economic well-being as well as an expanded set of outcomes adjusted for considerations of multiple testing bias. These tables include ITT estimates converted to effect size (ES), calculated as the ratio of the estimated difference between the high- and low-cash-gift groups divided by the standard deviation of the low-cash-gift group. Table 6 presents estimates transformed into marginal propensity to consume (MPC), capitalizing on the study’s experimental design by comparing MPCs from the cash gift with MPCs from all other sources of income, and further compares these MPCs with estimates derived from a nationally representative sample. Details about the construction of the study outcomes presented in the tables are provided in Appendix A11.

### A. Household income

Table 1 shows that household income, using a scaled measure of inflation-adjusted monthly non-cash-gift income plus the cash gift from the time of enrollment to the survey interview, increased by approximately $282.16 per month (details in Appendix Table A11), suggesting very little offset of other sources of income in light of receiving the unconditional cash transfer. Considering all sources of income, converted into monthly values, high-cash-gift families received slightly less earnings from other household members (−$44.56), government sources (−$8.80), and other sources (−$15.71); however, none of these differences in income statistically differed between the high- and low-cash-gift households. Maternal earnings were slightly, though not statistically significantly or substantively, higher in the high-cash-gift group (by $15.22) than in the low-cash-gift group.

The last four rows of Table 1 present impacts on the income-to-needs ratio, which is typically created by dividing pre-tax household income in the prior year by the corresponding federal poverty threshold for a given household size in the current year (e.g., $25,750 for a family of four with two children in 2019) such that a value of 1.0 indicates that a household is exactly at the federal poverty threshold. The high cash gift increased the income-to-needs ratio by 13.7, or by approximately 17% relative to the unadjusted income-to-needs ratio of low-cash-gift families. Using dichotomous categories of poverty status at the Wave 1 follow-up, high-cash-gift families were 7.1 percentage points less likely than low-cash-gift families to be at or below 100% of the federal poverty level and were 6.2 percentage points more likely than low-cash-gift families to be at 100%–200% of the federal poverty level. These estimates are robust to household membership assumptions using information about household membership from baseline or from the Wave 1 follow-up. At Wave 1, 94% of families—the vast majority—in both the high- and low-cash-gift groups were residing below 200% of the federal poverty threshold.

There were no statistically detectable differences in receipt of government assistance and benefits between high- and low-cash-gift families. Thus the cash gift did not (mechanically) crowd out government assistance as might be expected given the agreements in place to minimize risk of ineligibility due to the cash gift. At least two-thirds of the high-cash-gift families reported some receipt of social benefits at Wave 1 (as shown in Appendix A9, 64% reported receiving SNAP, 72% reported receiving WIC, and 66% reported receiving Medicaid). One exception is that high-cash-gift families were less likely to report receiving housing assistance (by 7.7 percentage points, or 33%) compared with low-cash-gift families, an effect that is statistically significant only among families in the New York City site.

### B. Maternal time in work and in child-enriching activities

Table 2 presents impacts on mothers’ time spent in paid work and time in developmentally enriching activities with the infant.^13^ At study entry, over 80% of mothers reported an intent to return to work or to start work in the next year (following the focal child’s birth). There were no statistically detectable differences in timing of mothers’ labor market entry or re-entry overall or their full-time employment after the child’s birth, nor in their employment at the time of the Wave 1 follow-up interview. Incidence of children’s experiences in nonparental care and out-of-pocket costs of childcare did not statistically differ between the high- and low-cash-gift families. Mothers were asked about the frequency of time spent reading, telling stories, playing with the child to build things, and participating in playgroups. The frequency of these activities, summed by activity in an index, was statistically higher in the high-cash-gift families relative to the low-cash-gift families. The activities with higher frequency among high-cash-gift families include book reading (ES=0.16) and telling stories (ES=0.13). High-cash-gift mothers were less likely to report “rarely or never” engaging in reading or telling stories, and were 6.5 percentage points (or 8%) more likely than low-cash-gift mothers to read books or tell stories a few times per week or more. Translating the categorical responses to a continuous measure of minutes spent on each activity,^14^ the impacts are equivalent to high-cash gift mothers increased time spent reading and telling stories to infants by approximately 2.7 minutes more per week, or a 10% increase.

### C. Child-specific expenditures

Table 3 presents impacts on child-related expenditures. High-cash-gift mothers spent $65.02 more in the 30 days prior to the survey interview on child-specific goods relative to expenditures by low-cash-gift mothers, an increase that was both economically (20%) and statistically (p<0.05) significant. The increased spending in the month prior to the survey interview included books ($7.38) and toys ($16.80), each of which was statistically significant at the 5% level, and clothes ($27.25) and diapers ($8.67), marginally statistically significant at the 10% level. (Note that the item on child-specific electronic goods or devices did not statistically differ between the high- and low-cash-gift groups.) Expenditures on these items for children in this age group are not readily available in nationally representative samples, as we describe below. However, these expenditure amounts are comparable to the $370 per month estimate for these same goods, including $17 per month on books and related early literacy and educational media, documented in a diverse sample of low-income families in New York City in 2004–2006 (Lugo-Gil and Yoshikawa 2006). Scaled to annual amounts, $780 of the $3,760, or approximately 21% of the cash gift differential, was allocated to children’s books, toys, clothing, diapers, and children’s electronic items/devices.

The high-cash-gift families not only increased total dollars allocated to these items but also increased the incidence of child-specific expenditures. High-cash-gift families were statistically more likely to purchase books by 11 percentage points (20% increase), toys by 3.5 percentage points (25% increase), and children’s clothing by 5.5 percentage points (6%). For the Wave 1 in-person pre-pandemic sample, interviewers observed high-cash-gift families’ homes as more likely to have children’s books (25%), compared with homes of low-cash-gift families (21%; ES=0.14; findings on this and related child-specific expenditures for the pre-pandemic sample are shown in Appendix Table A8). Table 3 further shows that high-cash-gift families were slightly more likely to purchase child-specific durable goods as measured via an index (marginally statistically significant); in particular, they were 7.6 percentage points more likely than low-cash gift families to have purchased a high chair since the birth of the infant.

### D. General core expenditures and household economic hardship

Table 4 presents impacts on a combined measure of general household expenditures and measures of economic hardship such as homelessness, as captured in survey reports (and includes family-wise adjustments). High-cash-gift families reported statistically higher expenditures by $152.17 on the combined measure of key consumption categories available through the survey, including expenditures on utilities, food,^15^ cable/internet/phone, out-of-pocket nonparental childcare, and the child-specific goods previously described. With the exception of expenditures on the collective bundle of child-focused goods (books, toys, diapers, clothing, and electronic items), none of the other expenditure categories statistically differed between the high- and low-cash-gift households, indicating a significant diversity in family expenditure allocations. As detailed earlier, steps were taken to minimize the impact on families’ eligibility for safety-net and related government assistance benefits due to receipt of the unconditional cash gift. Families may be funding consumption of goods such as food and housing through direct subsidization from government or private sources (e.g., food pantries). Additional unconditional cash may offer options to devote resources toward goods that are not subsidized by these sources.

The increase in the combined measure of available household general expenditures remains statistically significant when excluding expenditures on temptation goods such as alcohol, for which both levels and changes are small. Specifically, household expenditures on alcohol (approximately $4 on average in a typical week of the prior month) and cigarettes (one package in the prior month) did not statistically differ between the high- and low-cash-gift groups. This finding, coupled with the debit card data showing few transactions at liquor or tobacco stores or in casinos, offers little evidence in support of families allocating unconditional income toward drugs, alcohol, or related goods (see Yoo et al. 2022 for more).

Economists and social science scholars posit that increased income among families residing in poverty will improve material well-being and reduce hardships such as hunger and homelessness. We find no statistically detectable changes across a variety of pre-registered measures of material well-being or hardship. Maternal reports related to housing,^16^ health, or bill payment^17^ generally track national estimates, with no statistically detectable differences between the high- and low-cash-gift families (see Appendix Table A8 for item-level breakdowns of the indices presented in Table 4).

Impacts of the cash gift on food hardship are mixed. Paradoxically, maternal reports of food insecurity are higher in the high-cash-gift families than in the low-cash-gift families (0.14 ES), largely driven by higher reports of not being able to afford balanced meals. Fifteen percent of high-cash-gift families would be considered food insecure. As a point of comparison, 14.8% of households with children and 35.3% of households with income below the poverty line in 2020 were food insecure (USDA Economic Research Service 2021). Hardships related to housing quality, access to consumer durables (working heat or air conditioning, clothes washer or dryer), and neighborhood safety also did not statistically differ (at p<0.05) between the high- and low-cash-gift families. Further, impacts on these outcomes are not confounded by increased residential moves among high-cash-gift households.^18^ The high- and low-cash-gift families had no statistical differences in reported ownership of a working car or access to a smartphone or equivalent device or a tablet or desktop/laptop with an internet connection.

Whereas some empirical investigations show modest correlations between low income and various forms of material hardship (e.g., Mayer and Jencks 1989; Short 2005; Iceland and Bauman 2007); findings from two recent randomized controlled studies of lump sum cash transfers, and former welfare reform and related experiments, are mixed (see Jaroszewicz et al. 2022; Knox, Miller, and Gennetian 2000; Pilkauskas et al. 2022). Some reasons why poverty reduction might not translate into reductions in material hardship include the extent to which poverty measurement incorporates consumption of goods and services that may be funded by other sources such as wealth or debt (Meyer and Sullivan 2019) and whether the time horizon of income measurement corresponds with the timing of hardships. Food security can be short or intermittent, whereas other types of hardship, such as housing, can be longer. Further, not all income-poor households experience material hardship similarly, and income-poor households demonstrate a variety of strategies in juggling material hardships as a survival strategy (Edin and Shaefer 2015; Halpern-Meekin et al. 2015; Desmond 2016).

### E. Financial stress and subjective well-being

In this section, we turn to ITT estimates of mothers’ perspectives on financial strain and subjective well-being. Table 5 shows that the high cash gift marginally increased an index of economic stress (ES=0.10), particularly driven by the subcomponents of the scale related to subjective assessments of household spending totaling more than household income, an adjusted impact of 8 percentage points. There were no statistically detectable differences between the high- and low-cash-gift groups in reports of worry over expenses, incidence of setting aside emergency funds, or having the ability to cover a month of expenses without income.^19^ Although some mothers reported paying remittances, receiving the high cash gift did not statistically affect the amount of money given to support others not living in the household, as shown in Table 4 (e.g., payments or remittances for children or family members, not including loans or donations to charity). Given mothers’ expectations that the monthly cash gift would continue for at least 42 months (at the time of the Wave 1 survey), it will be important to assess in future waves whether planned intertemporal substitution of income enabled increases in certain types of expenditures in earlier years of a baby’s life with the knowledge of a future stream of cash gift income.

Several measures in the survey aimed to capture mothers’ overall subjective welfare (as distinct from psychological or mental health). Along this dimension, there were no statistically detectable differences in high- and low-cash-gift mothers’ happiness or on an 8-item index designed to capture maternal perceptions of agency and hope (e.g., meeting and pursuing goals, problem solving despite discouragement; details shown in Appendix Table A9).

### F. Propensity to consume child-related versus other household goods

We next formalize the empirical relationship between income and expenditures. Following an approach used in Del Boca and Flinn (1994), we estimate the following model:

(2) K=Zβ+γI+δ t+ε

where K indicates a category of goods across households, Z indicates baseline characteristics of the mother and household, as similarly included in the ITT estimates described earlier, I indicates any and all source of household income besides the monthly cash gift, and t indicates money from the monthly cash gift. If the child-related behavioral cues in the presentation of the cash gift are encouraging child-oriented spending behaviors that differ from spending behaviors from any and all other sources of income, then we would expect δ > γ for child-focused goods, such as books, diapers, and toys, and δ ≤ γ for other household goods, such as utilities and food.

We first generate covariate adjusted MPC models of total household income (I) for a sample of households with income at or below 200% of the official poverty line with at least one child under age 2 drawn from the Consumer Expenditure Survey over a comparable time period as the BFY Wave 1 survey. For the child-specific goods that allow comparison (toys and clothing), Table 6 shows that BFY families’ propensities to spend general household income on these goods are qualitatively similar to those of a nationally representative sample. The results for the nationally representative sample also show similar patterns of marginal propensity to consume on utilities and food as the BFY study sample, and a comparable negligible marginal propensity to consume on alcohol.

Findings from the BFY data further show a marginal propensity to consume 28% of the monthly cash gift on selected measured general household expenditures (with heterogeneity across line items) and a marginal propensity to consume approximately 20% of the monthly cash gift on selected measured child (nondurable) goods, specifically books, toys, diapers, and children’s clothes. The MPC on utilities and food from non-cash-gift sources of income are statistically similar to the MPC on these same goods from the cash gift (i.e. δ = γ). In contrast, the MPC on child-specific goods from the cash gift income is statistically larger than the MPC on child-specific goods from non-cash-gift sources of income (i.e. δ > γ).

## VII. Mothers’ views on the cash gift and time and money allocations

In-depth, semi-structured qualitative interviews have been instrumental in understanding how positive and negative income shocks, including those generated by policy, affect families (e.g., DeLuca et al. 2012; on Earned Income Tax Refunds, see Halpern-Meekin et al. 2015). We use such interviews with mothers to deepen our understanding of resource allocation decisions, particularly with regard to investments in children. Eighty mothers, 50 from New Orleans (25 in the high-cash-group; 25 in the low-cash-gift group) and 30 from the Twin Cities (15 in the high-cash-gift group; 15 in the low-cash-gift group), were recruited to participate in 1.5- to 2-hour interviews every 9 to 12 months over the follow-up period. The intention of these interviews is not to draw conclusions that are generalizable to all BFY participants, but rather to gain insights into the mothers’ perspectives and experiences related to time and money allocations that are difficult to capture in conventional quantitative data collection strategies.

First, mothers explicitly talk about mental earmarking of money for the baby. For example, Bianca,^20^ a 24-year-old Black mother of one from New Orleans in the high-cash-gift group, said, “I feel like that’s my baby’s money for the month. I make sure she gets her Pampers and wipes, everything she needs out of it.” Similarly, Jade, a 26-year-old Black mother of two from New Orleans in the low-cash-gift group, said, “It’s for the baby, so it’s for her. So I just spend it only on her…. I mean it’s just $20, just, you know…. Whatever; it helps.” The importance to mothers of having money mentally designated for their children was not taken lightly. Raven, a 31-year-old Black and American Indian mother in New Orleans in the high-cash-gift group, encapsulates this phenomenon. Raven says that without the BFY money, “I would not be able to give him more of what he wants…like, that money is strictly for him. Whatever he picks up, whatever he grabs, whatever he lacks is what that goes towards.”

Second, mental earmarking of money for the child did not always translate to spending on child-specific goods. For example, Isabella, a 30 year old, Latina mother of three, and her husband both saw the BFY dollars as “children’s money.” However, they differed in what they believed was the wisest way of using the money to contribute to their baby’s well-being. As Isabelle says “I can’t pause my kids, you know what I’m saying? Like if I need something in the house, that money will be spent for the fact that I need it in the house. It could be stuff to clean my house—it’s going to be spent.” In Isabella’s mind, taking care of immediate needs—like buying cleaning supplies in order to raise her son in a clean house—trumped consideration of the long-term, amorphous goals like saving that her husband preferred.

Third, mothers indicated how financial strain was top of mind. Many mothers found themselves in the maternity ward amid difficult financial circumstances. The cash gift was a wholly unexpected relief, and among many high-cash-gift mothers, it immediately went toward expenditures such as buying a car seat, paying an electricity bill, purchasing cleaning supplies, or purchasing related items to provide a safe home environment for the baby.^21^ Camille, a 26-year-old Black mother of two from New Orleans, used her first gift of $333 from BFY to get the lights turned back on and to buy two cans of formula for her new baby. Nina, a 26-year-old Black mother of four from New Orleans, recounted her reaction at learning she would receive $333 a month. She said, “I could have just cried because it was a total relief. Because first of all we went in the hospital flat broke. We was flat broke in the hospital.” With the first payment loaded onto the card, “We got food, a lot of food. We put food in the house. I even went and got the [older] kids a gift. That’s how happy I was. I was like, ‘Let me get the children something.’ So, I even got them a toy at the store. I got some cleaning supplies to make sure it was really sanitary for when I brought [my daughter] home.” Like Nina, multiple mothers described wanting to bring their children home from the hospital to clean houses, a gesture rich with the symbolism of a fresh start and an example of small, daily expenses that help parents feel that they are doing right by their children.

Fourth, some mothers viewed the cash gift as enabling new possibilities for the future. Tonya, a 42-year-old biracial mother of two from the Twin Cities, in the high-cash-gift group, felt a little worried that the BFY money was too good to be true, even after receiving it for a year, but nevertheless, she was excited about what the money could mean for her family’s financial future. “[I]f I could, say, save that, if I could just keep it there, it could be like an incredible start for the boys. So, right now I kind of act like it doesn’t exist, you know what I mean. I’ve gotten pretty good at living like real, real frugally. So, hopefully I can continue that.” Two other moms described using BFY money to invest in their side businesses. Camille, introduced above, sold hair bows, tutus, and birthday banners through her Instagram account. She used one month of the BFY money to buy the craft supplies she needed to continue making the items she sold. Alexandra, a 24-year-old Black mother of one in the Twin Cities, also saw the $333 in BFY money as enabling her to invest in a better future for herself and her family. With she and her husband both working, they were stressed and still barely getting by, so Alexandra began to stay home with their child and pursued a career as a writer; one month, she used the BFY money to invest in a writing course to support this effort. Mothers also described how the BFY money enabled them to handle their financial situations differently than they otherwise would, either not having to ask kin for assistance, or working fewer hours while still being able to cover expenses, though neither of these behaviors were captured as significant differences between gift groups in the quantitative survey data.

Finally, mothers conveyed how they viewed the cash gift as money independent from a government system, differently from other types of assistance. Over 90% of mothers reported receipt of some type of government assistance at study entry. Experiences with public benefit systems offer a context for mothers’ perspectives of the cash gift. For example, high-cash-gift mothers expressed fears that the money would stop prior to the pre-established 40 months (now extended to 52 months). Tonya, introduced above, had been receiving the BFY money for more than a year, yet she still described herself as “very wary…that it will be there.” Such reactions align with mothers’ expressions of simultaneous surprise and gratitude for the cash gift, using words like “blessing” or “thank you, God” when discussing first learning about the cash gift. This portrays the money’s arrival as akin to a miracle—and therefore a rare occurrence that cannot be explained or necessarily trusted to continue. Also, mothers described frustrating experiences with public benefit programs, with applications seemingly wrongfully denied or benefits simply not showing up as expected from one month to the next. Life had taught many that depending on anyone or anything other than themselves was a risk. To the extent that mothers felt unsure about the money’s regular arrival or contemplated the end of the cash-gift period looming on the horizon, taking on large expenses (such as higher rent^22^) may have seemed an overly risky gamble.

## VIII. Discussion and conclusion

This study reports on the causal impacts of an unconditional monthly cash transfer on family investments in infants among a racially and ethnically diverse sample of U.S. families residing in or near poverty at the time of the child’s birth. The high cash gift increased expenditures on child-specific goods such as books and toys even with post-gift income below 200% of poverty level. Compared with mothers who received the low cash gift, more high-cash-gift mothers reported engaging in child-specific early learning activities like reading books and telling stories with their children, without offsets in household earnings or statistically detectable changes in their own labor market participation or the incidence of nonparental care for the infant. Although a combined measure of household core expenditures measured in the survey including utilities, cable/internet/phone, and food was slightly higher among high-cash-gift versus low-cash-gift families, none of these individual spending categories statistically differed for the high- versus low-cash-gift families at conventional levels of statistical significance. That the cash gift had no statistically detectable impact on any one general household expenditure item suggests diversity in family spending from the unconstrained infusion of financial resources that we also see descriptively in the diversity of transactions from the debit card. The findings lend no support to the oft-cited critiques that unconditional money given to families residing in poverty will be spent on alcohol, cigarettes, and related temptation goods. The impact and higher marginal propensity to consume child-specific goods from the cash gift suggests mental earmarking of funds consistent with the child-related behavioral cues in the cash gift design.

Findings from the qualitative interviews echo the results from the survey-based expenditure analyses regarding heterogeneity in mothers’ allocations of the BFY cash gift. This offers a reminder that even as mothers overwhelmingly share the long-term goal of having happy, healthy children, the financial routes they pursue to that goal are varied. Just as Nina wants a clean house for her newborn’s sake, Camille dreams of building her business so she can pass it along to her kids. The unconditional nature of the BFY money offers families the option of choosing to allocate it in ways that align with their specific situations and values and shifting income circumstances.

This study demonstrates how a predictable unconditional monthly cash transfer can generate direct investments in time and money inputs during the first year of a child’s life, thus complementing an extensive empirical literature on the impacts of earmarked social investments that have in turn been shown to improve children’s development (Almond, Currie, and Duque 2018), including increased birth weight (Almond, Hoynes, and Schanzenbach 2011; Hoynes, Miller, and Simon 2015), higher school achievement (Duncan, Morris, and Rodrigues 2011; Dahl and Lochner 2012, 2017), reductions in juvenile crime and psychiatric disorders (Akee et al. 2010; Anders, Barr, and Smith forthcoming; Barr and Smith 2021), and higher earnings and improved cardiovascular health in adulthood (Aizer et al. 2016; Hoynes, Schanzenbach, and Almond 2016; Chetty, Hendren, and Katz 2016).^23^ The developmental psychology literature finds that the early learning activity inputs examined in this study are predictors of children’s language and literacy development (Noble et al. 2019) that are, in turn, positively associated with schooling outcomes (Wade 2004; Cook, Roggman, and Boyce 2011; Hardaway et al. 2020). Early looks at the impact of the high-cash gift on infant brain functioning among a subsample of families (n=440) in this same RCT indeed showed positive, substantive impacts (Troller-Renfree et al. 2022), and a more complete appraisal of cumulative impacts on child development is underway.

## Figures and Tables

**Table 1.** Impacts on Household Economic Resources

	Low-cash-gift group mean	High-cash-gift group mean	OLS	OLS w/covariates	Effect size	p-value	N
Household average monthly income including BFY cash gift	1,917.237	2,128.724	212.835 (94.009)	282.162 (85.848)	0.20	0.001	931
Mother’s average monthly earned income	596.615	600.961	4.063 (45.157)	15.224 (44.197)	0.02	0.731	922
Average other monthly household income: partner/other earned, government, and all other income not including BFY gift	1,444.666	1,257.085	−175.022 (100.276)	−88.268 (94.403)	−0.05	0.350	880
Average monthly spouse and other household member’s earned income	1,023.403	924.751	−89.763 (77.149)	−44.564 (70.935)	−0.04	0.530	892
Average monthly household government income	186.485	171.176	−15.076 (21.182)	−8.795 (21.443)	−0.03	0.682	915
Average monthly household all other income	67.676	50.881	−16.786 (9.805)	−15.713 (9.830)	−0.10	0.110	923
[preR] Social Services Receipt Index	2.874	2.888	0.014 (0.110)	−0.080 (0.104)	−0.05	0.443	931
Income-to-needs ratio (including cash gift)	0.799	0.892	0.094 (0.039)	0.137 (0.037)	0.23	0.000	931
[preR] Below 100% FPL incl. cash gift (Income-to-needs <1)	0.714	0.658	−0.057 (0.031)	−0.095 (0.029)	−0.21	0.001	931
100% to <200% FPL incl. cash gift (Income-to-needs 1 to <2)	0.232	0.282	0.051 (0.029)	0.082 (0.029)	0.19	0.005	931
≥200% FPL incl. cash gift (Income-to-needs ≥2)	0.055	0.060	0.005 (0.015)	0.013 (0.016)	0.06	0.420	931

[preR] indicates that the outcome was pre-registered; see socialscienceregistry.org/trials/3262. All income values adjusted to 2019. Detailed description of outcomes are available in Appendix Table A11.

Standard errors in parentheses. P-values are for coefficient on treatment from OLS of outcome on treatment and covariates with site fixed effects. ITT estimates of items in Social Services Receipt Index provided in Appendix Table A9. Covariates from baseline survey: Mother’s age, Completed schooling, Household income, Net worth, General health, Mental health, Race and ethnicity, Marital status, Number of adults in the household, Number of other children born to the mother, Smoked during pregnancy, Drank alcohol during pregnancy, Father living with the mother, Child’s sex, Birth weight, Gestational age at birth. Other covariates: Phone interview, child age at interview (in months).

**Table 2.** Impacts on Maternal Time Use

	Low-cash-gift group mean	High-cash-gift group mean	Coefficient	Coefficient w/covariates	Effect size	p-value	N
***Mother’s Time in Paid Work***							
[preR] Months to labor market reentry from birth	3.699	3.628	−0.028 (0.086)	−0.025 (0.092)	N/A	0.783	931
[preR] Months to full-time labor market reentry from birth	3.674	3.737	−0.091 (0.118)	0.016 (0.126)	N/A	0.896	931
Mother working in paid job at time of Wave 1 follow-up	0.453	0.410	−0.043 (0.033)	−0.045 (0.033)	−0.09	0.179	931
Mother’s total hours worked per week at all jobs	18.489	17.426	−1.217 (1.450)	−1.999 (1.486)	−0.10	0.179	749
[preR] Mother’s education and training attainment indicator	0.261	0.272	0.010 (0.029)	0.014 (0.030)	0.03	0.650	931
***Mother’s Time with Child and Child Time in Nonparental Care***							
[preR] Any time in a childcare or day care center (last year)	0.274	0.298	0.024 (0.030)	0.022 (0.030)	0.05	0.462	929
Anyone other than parents looked after baby last week	0.460	0.455	−0.004 (0.033)	0.000 (0.034)	0.00	0.992	930
[preR] Parent-Child Activities Index	10.285	10.780	0.498 (0.175)	0.438 (0.180)	0.16	0.015	929
Read books together	2.867	3.016	0.150 (0.061)	0.154 (0.064)	0.16	0.016	929
Tell stories	2.771	2.942	0.172 (0.070)	0.144 (0.072)	0.13	0.046	929
Play to build things	3.179	3.312	0.134 (0.069)	0.105 (0.072)	0.10	0.147	929
Play groups	1.468	1.510	0.042 (0.056)	0.036 (0.058)	0.04	0.533	929
Reads books or tells stories a few times a week or more	0.808	0.874	0.067 (0.024)	0.065 (0.025)	0.16	0.009	929

[preR] indicates that the outcome was pre-registered; see socialscienceregistry.org/trials/3262. All income values adjusted to 2019. Detailed description of outcomes are available in Appendix Table A11.

Standard errors in parentheses. P-values are for coefficient on treatment from OLS of outcome on treatment and covariates with site fixed effects.

Covariates from baseline survey: Mother’s age, Completed schooling, Household income, Net worth, General health, Mental health, Race and ethnicity, Marital status, Number of adults in the household, Number of other children born to the mother, Smoked during pregnancy, Drank alcohol during pregnancy, Father living with the mother, Child’s sex, Birth weight, Gestational age at birth. Other covariates: Phone interview, child age at interview (in months). Mean for time to work outcomes not reported as not all mothers returned to work. Due to a survey administration error, individuals surveyed early in Wave 1 data collection who responded “No” to “Do you currently work for pay?” were not asked the follow-up questions such as “Are you currently self-employed?” or about hours worked, resulting in the smaller N of 749 for hours worked. This affects approximately 168 observations.

Coefficients and p-values for whether the mother was in paid work and the child activity index outcomes are from OLS. Coefficients for the time to work outcomes are from Cox-proportional hazard models with time variable for mothers that did not return to (full-time) set to the time of the Wave 1 interview or 21 months if the Wave 1 interview was conducted after 21 months because mothers were only asked about life events for 21 months post birth. Both control for all baseline covariates with site fixed effects.

**Table 3.** Impacts on Child-Related Expenditures

	Low-cash-gift group mean	High-cash-gift group mean	OLS	OLS w/covariates	Effect size	p-value	N
[preR] Amount spent on childcare in average month (household)	188.37	226.66	38.43 (26.89)	32.08 (25.94)	0.08	0.227	931
[preR] Child-focused Expenditure Index (amount in last 30 days)	313.35	363.70	50.47 (22.09)	65.02 (23.07)	0.23	0.005	931
Money spent on books (past 30 days)	13.10	18.89	5.77 (1.71)	7.38 (1.66)	0.32	0.000	931
Money spent on toys (past 30 days)	69.99	83.98	13.85 (6.33)	16.80 (6.62)	0.16	0.01	931
Money spent on clothes (past 30 days)	144.30	166.32	22.17 (15.71)	27.25 (16.39)	0.17	0.10	931
Money spent on diapers (past 30 days)	71.74	78.39	6.77 (4.83)	8.67 (4.99)	0.15	0.08	931
Money spent on children’s electronics (past 30 days)	14.22	16.11	1.91 (4.04)	4.92 (4.67)	0.10	0.29	931
[preR] Purchases for Child Since Birth Index	4.80	4.91	0.12 (0.13)	0.24 (0.14)	0.12	0.07	931
Books purchased	0.84	0.84	−0.01 (0.03)	0.006 (0.026)	0.02	0.81	930
Crib purchased	0.70	0.70	0.004 (0.03)	0.02 (0.032)	0.04	0.57	931
Car seat purchased	0.83	0.85	0.02 (0.02)	0.025 (0.025)	0.06	0.32	931
High chair purchased	0.59	0.64	0.05 (0.03)	0.076 (0.032)	0.15	0.02	930
Number of child safety devices purchased (0–4 possible)	1.84	1.89	0.05 (0.09)	0.12 (0.09)	0.09	0.20	931
Any child safety devices purchased	0.80	0.82	0.02 (0.03)	0.039 (0.027)	0.10	0.15	931

[preR] indicates that the outcome was pre-registered; see socialscienceregistry.org/trials/3262. All income values adjusted to 2019. Detailed description of outcomes are available in Appendix Table A11.

Standard errors in parentheses. P-values are for coefficient on treatment from OLS of outcome on treatment and covariates with site fixed effects.

Covariates from baseline survey: Mother’s age, Completed schooling, Household income, Net worth, General health, Mental health, Race and ethnicity, Marital status, Number of adults in the household, Number of other children born to the mother, Smoked during pregnancy, Drank alcohol during pregnancy, Father living with the mother, Child’s sex, Birth weight, Gestational age at birth. Other covariates: Phone interview, child age at interview (in months). Child-focused Expenditure Index is the sum of expenditures on books, toys, clothes, diapers, and electronics. Child safety devices included outlet cover, safety latch, safety gate, and smoke detector.

**Table 4.** Impacts on Other Household Expenditures and Investments

	Low-cash-gift group mean	High-cash-gift group mean	OLS	OLS w/covariates	Effect size	p-value	Wyoung adj p-value	N
***Expenditures, average month***								
Household expenditures, including child-focused	1,791.926	1,931.043	138.544 (55.553)	152.169 (55.642)	0.18	0.006	0.004	931
Household non-child-focused expenditures, including childcare	1,478.574	1,567.348	88.078 (47.050)	87.149 (46.836)	0.12	0.063	0.071	931
Amount spent on home utilities	217.167	231.257	13.219 (10.592)	13.349 (10.641)	0.08	0.210	0.211	931
Amount spent on home cable, internet, and phone	155.831	157.406	1.794 (7.105)	3.933 (7.089)	0.04	0.579	0.595	931
Amount spent on food	718.903	738.316	19.471 (26.337)	9.582 (26.717)	0.02	0.720	0.719	931
Amount spent eating out	187.629	203.414	15.549 (14.910)	27.393 (15.064)	0.12	0.069	0.064	931
Amount spent to support others	7.271	6.798	−0.482 (1.825)	0.183 (1.978)	0.01	0.926	0.924	931
Amount spent on alcohol in average week	3.400	3.496	0.091 (0.649)	0.632 (0.685)	0.07	0.357	0.363	931
Packs of cigarettes purchased in average week	1.103	0.987	−0.123 (0.295)	0.053 (0.249)	0.01	0.832	0.844	927
***Household material needs and hardship***								
Mother has been homeless since baby’s birth	0.068	0.063	−0.005 (0.016)	0.006 (0.016)	0.02	0.718	0.656	930
[preR] Food Insecurity Index	1.209	1.491	0.281 (0.115)	0.230 (0.119)	0.14	0.054	0.256	929
[preR] Perceptions of Neighborhood Safety Index	4.382	4.186	−0.198 (0.089)	−0.170 (0.094)	−0.13	0.071	0.942	926
[preR] Housing Quality Index	6.336	6.306	−0.031 (0.145)	−0.035 (0.150)	−0.02	0.813	0.929	930
[preR] Excessive residential mobility (≥3 times)	0.060	0.057	−0.003 (0.016)	0.010 (0.016)	0.04	0.517	0.711	930

[preR] indicates that the outcome was pre-registered; see socialscienceregistry.org/trials/3262. All income values adjusted to 2019. Detailed description of outcomes are available in Appendix Table A11.

Standard errors in parentheses. P-values are for coefficient on treatment from OLS of outcome on treatment and covariates with site fixed effects. ITT estimates of items for the Food Insecurity Index, Perceptions of Neighborhood Safety Index, and Housing Quality Index are presented in Appendix Table A9.

Covariates from baseline survey: Mother’s age, Completed schooling, Household income, Net worth, General health, Mental health, Race and ethnicity, Marital status, Number of adults in the household, Number of other children born to the mother, Smoked during pregnancy, Drank alcohol during pregnancy, Father living with the mother, Child’s sex, Birth weight, Gestational age at birth. Other covariates: Phone interview, child age at interview (in months).

**Table 5.** Impacts on Economic Stress and Subjective Well-being

	Low-cash-gift group mean	High-cash-gift group mean	OLS	OLS w/covariates	Effect size	p-value	Wyoung adj p-value	N
[preR] Index of Economic Stress	2.678	2.924	0.250 (0.125)	0.184 (0.128)	0.10	0.153	0.148	930
Economic Stress Index: Financial worry	1.916	2.123	0.211 (0.082)	0.157 (0.083)	0.13	0.060	0.070	929
Worry always or very frequently about expenses	0.389	0.449	0.061 (0.033)	0.045 (0.034)	0.09	0.175	0.173	930
Household spent more than income	0.441	0.521	0.083 (0.033)	0.081 (0.034)	0.16	0.019	0.019	917
Haven’t set aside emergency funds	0.718	0.713	−0.004 (0.030)	−0.025 (0.030)	−0.06	0.400	0.438	930
Could not cover a month of expenses without income	0.376	0.445	0.069 (0.033)	0.054 (0.034)	0.11	0.115	0.146	924
Economic Stress Index: Hardships	0.766	0.802	0.034 (0.068)	0.020 (0.070)	0.02	0.773	0.759	929
Missed a rent or mortgage payment	0.208	0.245	0.038 (0.028)	0.032 (0.029)	0.08	0.263	0.264	927
Missed utility payment	0.302	0.325	0.022 (0.031)	0.015 (0.032)	0.03	0.644	0.636	928
Shut off utilities	0.093	0.073	−0.021 (0.018)	−0.022 (0.019)	−0.08	0.243	0.240	928
Mother has been evicted since baby’s birth	0.068	0.060	−0.008 (0.016)	−0.001 (0.016)	−0.00	0.947	0.956	930
Mother or child missed medical/dental care in last year	0.095	0.099	0.004 (0.020)	−0.002 (0.020)	−0.01	0.931	0.919	929
***Maternal subjective well-being***								
[preR] HOPE Maternal Agency 8-item scale	31.718	31.321	−0.389 (0.303)	−0.349 (0.310)	−0.08	0.261	0.424	929
[preR] Maternal Global Happiness mean	2.236	2.254	0.018 (0.047)	0.008 (0.047)	0.01	0.866	0.866	925

[preR] indicates that the outcome was pre-registered; see socialscienceregistry.org/trials/3262. All income values adjusted to 2019. Detailed description of outcomes are available in Appendix Table A11.

Standard errors in parentheses. P-values are for coefficient on treatment from OLS of outcome on treatment and covariates with site fixed effects. ITT estimates of HOPE Maternal Agency items are provided in Appendix Table A9. Covariates from baseline survey: Mother’s age, Completed schooling, Household income, Net worth, General health, Mental health, Race and ethnicity, Marital status, Number of adults in the household, Number of other children born to the mother, Smoked during pregnancy, Drank alcohol during pregnancy, Father living with the mother, Child’s sex, Birth weight, Gestational age at birth. Other covariates: Phone interview, child age at interview (in months).

**Table 6.** Marginal Propensity to Consume, Consumer Expenditures Survey (CES) and Baby’s First Years Study Wave 1 Sample

	CES^1^		Baby’s First Years^2^				
	Income	P-value	Income	Gift	P-value income	P-value gift	P-value difference
Amount spent on childcare in average month	0.001 (0.000)	0.048	0.020 (0.011)	0.104 (0.083)	0.068	0.211	0.310
Child-focused Expenditure Index, last 30 days			0.026 (0.008)	0.209 (0.073)	0.001	0.004	0.014
Money spent on books	0.004 (0.003)	0.186	0.001 (0.001)	0.024 (0.005)	0.180	0.000	0.000
Money spent on toys	0.009 (0.005)	0.07	0.008 (0.003)	0.054 (0.021)	0.011	0.010	0.033
Money spent on clothes	0.002 (0.001)	0.107	0.013 (0.004)	0.088 (0.052)	0.003	0.093	0.158
Money spent on diapers	0.001 (0.001)	0.237	0.003 (0.002)	0.028 (0.016)	0.185	0.081	0.118
Money spent on electronics			0.002 (0.001)	0.016 (0.015)	0.288	0.289	0.337
Amount spent on home utilities in average month	0.012 (0.003)	0.001	0.012 (0.005)	0.043 (0.034)	0.008	0.199	0.366
Amount spent on home cable, internet, and phone in average month			0.013 (0.003)	0.013 (0.022)	0.000	0.551	0.994
Amount spent on food by month	0.023 (0.005)		0.015 (0.011)	0.032 (0.085)	0.147	0.712	0.849
Amount spent eating out in average month	0.005 (0.001)		0.026 (0.006)	0.089 (0.047)	0.000	0.060	0.189
Amount spent on alcohol in average week	0.000 (0.000)	0.733	0.001 (0.000)	0.002 (0.002)	0.000	0.335	0.662

Consumer Expenditure Survey drawn from 2018Q3–2020Q3 overlapping with BFY Wave 1 data of families below 200% FPL with a child <2 years old. Estimates on diapers, books, toys, and cigarettes/tobacco come from the diary survey of 3,000 households per quarter. Information on all other expenditure categories come from the interview survey drawing from 6,000 households per quarter. Estimates adjust age of mother, number of children in household, race/ethnicity of mother, marital status, total household size, and state/year/quarter fixed effects. Standard errors in parentheses, clustered at household level.

2 Estimates adjust for covariates as described in Tables 1–5. Coefficients derived from OLS estimates of monthly expenditure in each row on monthly average total household income other than gift; and gift ($333 or $20. The p-value difference is from Wald test of equality of coefficient on income and on gift.

## Footnotes

1 The limited evidence base on the impacts of unconditional cash in the U.S. includes quasi-experimental studies in the U.S. from Akee et al. (2010) on casino lottery payments to Eastern Cherokee families, Aizer et al. (2016) on pensions to widowed mothers, and studies of unconditional payments from the Alaska permanent fund (e.g., Jones and Marinescu, forthcoming).

2 Upon study enrollment, mothers were informed that the monthly cash gift would be available for 42 months, as the study was initially envisioned as providing supplemental income through the first three years of the child’s life with capstone in-person child development data collection planned at child age 3 years. In response to the pandemic and disruption of in-person data collection, subsequent successful fundraising supported extending the cash gift to 52 months with in-person child development capstone data collection at child age 4 years. All details of the study design can be found at babysfirstyears.com or in the AEA registration at https://www.socialscienceregistry.org/trials/3262/history/95070.

3 Examinations of the empirical relationship between circumstances of poverty and adult cognition include Mani et al. (2013); Schofield and Venkataramani (2021); and Shah, Mullainathan, and Shafir (2012).

4 Perceptions of current versus future enjoyment, for example, affect assessments of investments in expenditures including necessities such as food (Banerjee and Duflo 2019, chapter 4). Myopia, or present bias, may interfere with valuation of future benefits, and expectations of future returns on general household material goods and needs may differ from valuation of returns on child-related goods such as books and toys (Mayer et al. 2018). Assessment of risks may also be related to present bias and thus vary across types of expenditures (Edin and Shaefer 2015; Morduch and Schneider 2017). Budget items like medical debt may be accumulated despite high interest rates, whereas other items such as cell phone payments are prioritized. Parent preferences may also be shaped by habit formation and updating of information through new knowledge or learned behavior (Cunha, Elo, and Culhane 2013). For example, parental updating of beliefs about theimportance of kindergarten attendance reduced chronic absenteeism (Robinson et al. 2018). Finally, social norms and perceptions may affect relative evaluations that shape spending, as has been documented in analyses of data from the U.K., where low-income families dedicated a greater portion of their income to children in an effort to “catch up” with their affluent counterparts (Kornrich and Furstenberg 2013).

5 The cash transfer is a gift available through charitable organizations and is not taxable due to its source and unconditional nature. At study entry and upon consent to participate in the randomized controlled study, mothers received a letter detailing implications for taxes and receipt of other government benefits. See Gennetian et al. (forthcoming) for more about the cash gift design. A picture of the debit card and information for study participants are available at https://www.4mybabycard.com/.

6 More information is available via AEA pre-registration: https://www.socialscienceregistry.org/trials/3262.

7 The University of Michigan Institute for Social Research collected baseline and Wave 1, 2, and 3 follow-up data.

8 The Wave 1 follow-up survey included a module of month-by-month reporting of mother’s employment, use of childcare, and breastfeeding. A full analysis of the monthly calendar data tracking the co-occurrence of these pre-registered outcomes is beyond the scope of this study.

9 Less than 10% of transactions show up more than once on exactly the same date, for the same amount, with the same transaction category. Removing duplicates results in approximately 57,000 transactions. While some duplicates may be legitimate (e.g., a mother withdrawing cash at ATM twice because of limits on the amount that can be drawn at one time), we decided to run analyses with and without duplicates. Conclusions do not substantively vary when we compare analyses with and without duplicates.

10 Baseline characteristics of these 16 mothers: 11 Hispanic; 6 single, 6 married; 9 high school/GED, 6 less than high school; 5 with focal child as first birth; and 13 with biological father residing in the household at child’s birth.

11 Mothers are free to call Mastercard regarding any difficulties, or they can call customer service at a 4MyBaby hotline (hosted and managed by research assistants and students at Teachers College, Columbia University). Approximately 2,550 calls were received between study entry and the end of the Wave 1 data collection, with 45% of the BFY mothers calling at least once, primarily seeking clarity about the study or the debit card.

12 There is baseline equivalence across groups with respect to reasons for nonresponse related to the study focal child being deceased, study focal child no longer being in mother’s custodial care, and mother being incarcerated. There were more refusals to participate in the Wave 1 follow-up among low-cash-gift mothers (n=6) than among high-cash-gift mothers (n=0; small difference is statistically significant at p<0.05), and more low-cash-gift mothers (n=38) were not found or were not able to be contacted for the Wave 1 follow-up than high-cash-gift mothers (n=12; small difference is statistically significant at p<0.05).

13 Sleep, both quantity and quality, is increasingly recognized as an important element of time use, affecting economic productivity as well as psychological well-being, cognition, and parenting (Bessone et al. 2021). At the Wave 1 follow-up, high-cash-gift mothers reported similar overall ratings on quality of sleep, feeling tired, and difficulty of falling asleep as low-cash-gift mothers, shown in Appendix Table A7.

14 Drawing on estimates from Kalil et al. (2012) for maternal time spent with children on teaching and play derived from the American Time Use Survey, we translate the the likert scale response categories into minutes. If a BFY mother responds rarely, we assign 0 minutes; if a mother responds a few times a month, we assign the value for 1 weekend day; if a mother responds weekly, we assign the value for 3 weekend days; if a mother responds daily we assign 7 days a week. We assign these values in minutes to each BFY mother by level of baseline education (less than high school, high school, some college, college or more). We then re-run the ITT estimates for these new transformations of the reading, telling stories, and each playing task item. Note that while the mean values may shift, the ITT conclusions are qualitatively similar under different assignment assumptions for “a few times a month” and “weekly.”

15 In the Wave 1 follow-up survey, the subset of mothers who reported receiving SNAP but also reported that no one else in the household was receiving SNAP were not provided the prompt to exclude food stamp benefits in their reporting of the amount spent on food per week. Thus, for this subset of mothers, food purchased through SNAP benefits might be included in overall spending on food. While this affects the interpretation of overall expenditures on food, it should not affect the impact estimate since mothers and households in high-cash-gift families do not statistically differ in reported receipt of SNAP benefits.

16 Approximately one-fifth of mothers reported missing rent or a mortgage payment (four times higher than national estimates). Nearly 7% of mothers reported being evicted (close to average eviction filing rates in 2016; Desmond 2016), and 8% reported being homeless or in a group shelter since the baby’s birth.

17 For example, utilities comprise up to 20% of net income of households in the lowest income decile, with one-third of U.S. households reporting difficulties meeting energy needs by either forgoing other necessities to meet energy bills, receiving disconnection notices, or keeping a home at an unhealthy or unsafe temperature. Six million households nationally received heating assistance alone, with an average benefit of $371 per household annually, covering about half of each family’s heating bills. A third or more of mothers reported missing a utility payment (slightly higher than estimates for the general population), with mothers reporting spending on average of $228 in utilities and $160 in cable payments a month. Less than 10% of mothers reported having utilities cut off in the prior 12 months, whereas closer to 40% reported having cable cut off.

18 In our study, 40% of mothers reported at least one residential move since the time of baby’s birth, and 6% report moving three or more times (by comparison, 10% of families in the Moving to Opportunity study moved three or more times over the 10- to 15-year follow-up; Ludwig et al. 2013). Nearly two-thirds of mothers reported that their housing condition is good or excellent.

19 Some insights may be gleaned on these subjective assessments of financial strain by comparing reports at the Wave 1 follow-up with reports at study entry on these same items. Approximately one-third of mothers reported financial worry at study entry and 35% reported spending more in total than their income. Thus, reports of financial worry and spending more in total than their income increased for both groups by the time of the Wave 1 follow-up, though slightly more for mothers in the high-cash-gift group. We also note that although the survey question indicated to count the cash gift as income with respect to the item on spending more, we cannot rule out mothers’ mental earmarking of the cash gift differently from their perception of counting income in their reporting of this item. Mothers in both groups were less likely to report not being able to cover expenses without income at the Wave 1 follow-up relative to study entry (from 48% to 38% unadjusted in the low-cash-gift group and from 45% to 54% in the high-cash-gift group). The difference, however, between the high- and low-cash-gift group at study entry, and at the Wave 1 follow-up, is statistically similar (with a difference of ES 0.11).

20 In line with common practice, we use pseudonyms to protect mothers’ identities

21 For example, while some states prohibit utility shutoffs for households with infants or children, Louisiana does not (U.S. Department of Health and Human Services n.d.).

22 Other research indicates that families consider multiple factors when moving, including being open to neighborhoods that are unsafe in order to access enough space within an apartment and have access to necessary public transit, as well as to find landlords who are willing to accept them as tenants (Rosenblatt and DeLuca 2012). As in other domains of life, therefore, families’ residential choices may be more constrained than their finances alone would suggest. We could anticipate that such factors would limit the impact of the additional BFY dollars in families’ abilities to make particular purchases, especially in arenas in which market and non-market forces may constrain supply or quality (e.g., housing, childcare).

23 Comparable studies in other contexts include those of Milligan and Stabile (2011), who found benefits of the Canadian child benefit on children’s math achievement, and Black et al. (2014), who found net income benefits of Norway’s childcare subsidy on children’s academic performance in junior high school. In contrast, Cesarini et al. (2016) examined the impact of wealth shocks from Sweden’s lottery and concluded that wealth is not a major source of the observed relationships between child developmental outcomes and household income in countries with robust social safety nets such as Sweden’s.

## References

[R1] Aizer Anna, and Cunha Flávio. 2012. “The Production of Human Capital: Endowments, Investments and Fertility.” NBER Working Paper 18429.

[R2] Aizer Anna, Eli Shari, Ferrie Joseph, and Lleras-Muney Adriana. 2016. “The Long-Run Impact of Cash Transfers to Poor Families.” American Economic Review 106 (4): 935–71. 28713169 10.1257/aer.20140529 PMC5510957

[R3] Akee Randall K. Q., Copeland William E., Keeler Gordon, Angold Adrian, and Costello E. Jane. 2010. “Parents’ Incomes and Children’s Outcomes: A Quasi-Experiment Using Transfer Payments from Casino Profits.” American Economic Journal: Applied Economics 2 (1): 86–115. 20582231 10.1257/app.2.1.86 PMC2891175

[R4] Akerlof George and Kranton Rachel. 2010. Identity Economics: How our Identities Shape Our Work, Wages and Well-Being. Princeton, NJ: Princeton University Press.

[R5] Algan Yann, Beasley Elizabeth, Sylvana Côté Jungwee Park, Tremblay Richard E., and Vitaro Frank. 2022. “The Impact of Childhood Social Skills and Self-Control Training on Economic and Noneconomic Outcomes: Evidence from a Randomized Experiment Using Administrative Data.” American Economic Review, 112 (8): 2553–79.

[R6] Almond Douglas, Currie Janet, and Duque Valentina. 2018. “Childhood Circumstances and Adult Outcomes: Act II.” Journal of Economic Literature 56 (4): 1360–1446.

[R7] Almond Douglas, Hoynes Hilary W., and Schanzenbach Diane Whitmore. 2011. “Inside the War on Poverty: The Impact of Food Stamps on Birth Outcomes.” Review of Economics and Statistics 93 (2): 387–403.

[R8] Altmejd Adam, Andrés Barrios-Fernández Marin Drlje, Goodman Joshua, Hurwitz Michael, Kovac Dejan, Mulhern Christine, Neilson Christopher, and Smith Jonathan. 2021. “O Brother, Where Start Thou? Sibling Spillovers on College and Major Choice in Four Countries.” Quarterly Journal of Economics 136 (3): 1831–86.

[R9] Anders John, Barr Andrew C., and Smith Alex. Forthcoming. “The Effect of Early Childhood Education on Adult Criminality: Evidence from the 1960s through 1990s.” American Economic Journal: Economic Policy.

[R10] Attanasio Orazio P., and Kaufmann Katja M.. 2009. “Educational Choices, Subjective Expectations, and Credit Constraints.” NBER Working Paper 15087.

[R11] Attanasio Orazio P., and Lechene Valérie. 2002. “Tests of Income Pooling in Household Decisions.” Review of Economic Dynamics 5 (4): 720–48.

[R12] Banerjee Abhijit V., and Duflo Esther. 2019. Good Economics for Hard Times. New York: Hachette.

[R13] Barr Andrew, and Smith Alexander A.. 2021. “Fighting Crime in the Cradle: The Effects of Early Childhood Access to Nutritional Assistance.” Journal of Human Resources. Published ahead of print, April 13. 10.3368/jhr.58.3.0619-10276R2.

[R14] Becker Gary S., and Tomes Nigel. 1976. “Child Endowments and the Quantity and Quality of Children.” Journal of Political Economy 84 (4, pt. 2): S143–S162.

[R15] Benhassine Najy, Devoto Florencia, Duflo Esther, Dupas Pascaline, and Pouliquen Victor. 2015. “Turning a Shove into a Nudge? A ‘Labeled Cash Transfer’ for Education.” American Economic Review: Economic Policy 7 (3): 86–125.

[R16] Bessone Pedro, Rao Gautam, Schilbach Frank, Schofield Heather, Toma Mattie, 2021. “The Economic Consequences of Increasing Sleep Among the Urban Poor.” The Quarterly Journal of Economics 136 (3): 1887–1941. 34220361 10.1093/qje/qjab013 PMC8242594

[R17] Bitler Marianne P., Hoynes Hilary W., and Schanzenbach Diane Whitmore. 2020. “The Social Safety Net in the Wake of COVID-19.” Brookings Papers on Economic Activity 2020 (2): 119–58.

[R18] Black Sandra E., Devereux Paul J., Løken Katrine V., and Salvanes Kjell G.. 2014. “Care or Cash? The Effect of Child Care Subsidies on Student Performance.” The Review of Economics and Statistics 96 (5): 824–33.

[R19] Browning Martin, Chiappori Pierre-André, and Weiss Yoram. 2014. Economics of the Family. New York: Cambridge University Press.

[R20] Caucutt Elizabeth M., and Lochner Lance. 2021. “Early and Late Human Capital Investments, Borrowing Constraints, and the Family.” Journal of Political Economy 128 (3): 1065–1147.

[R21] Cesarini David, Lindqvist Erik, Östling Robert, and Wallace Björn. 2016. “Wealth, Health, and Child Development: Evidence from Administrative Data on Swedish Lottery Players.” The Quarterly Journal of Economics 131 (2): 687–738.

[R22] Chetty Raj, Hendren Nathaniel, and Katz Lawrence F.. 2016. “The Effects of Exposure to Better Neighborhoods on Children: New Evidence from the Moving to Opportunity Experiment.” American Economic Review 106 (4): 855–902. 29546974 10.1257/aer.20150572

[R23] Cook Gina A., Roggman Lori A., and Boyce Lisa K.. 2011. “Fathers’ and Mothers’ Cognitive Stimulation in Early Play with Toddlers: Predictors of 5th Grade Reading and Math.” Family Science 2 (2): 131–45.

[R24] Conti Gabriella, James J Heckman Rodrigo Pinto. 2016. The Effects of Two Influential Early Childhood Intervention Programs on Health and Behavior. The Economic Journal 125(596): F28–F65. 10.1111/ecoj.12420 PMC5331750 28260805

[R25] Cunha Flávio, Elo Irma, and Culhane Jennifer. 2013. “Eliciting Maternal Expectations about the Technology of Cognitive Skill Formation.” NBER Working Paper 19144.

[R26] Cunha Flavio, and Heckman James. 2007. “The Technology of Skill Formation.” American Economic Review 97 (2): 31–47.

[R27] Dahl Gordon B., and Lochner Lance. 2012. “The Impact of Family Income on Child Achievement: Evidence from the Earned Income Tax Credit.” American Economic Review 102 (5): 1927–56.

[R28] Dahl Gordon B., and Lochner Lance. 2017. “The Impact of Family Income on Child Achievement: Evidence from the Earned Income Tax Credit: Reply.” American Economic Review 107 (2): 629–31.

[R29] Boca Del, Daniela Christopher Flinn, and Wiswall Matthew. 2014. “Household Choices and Child Development.” Review of Economic Studies 81 (1): 137–85.

[R30] Boca Del Daniela, and Flinn Christopher J.. 1994. “Expenditure Decisions of Divorced Mothers and Income Composition.” Journal of Human Resources 29 (3): 742–61.

[R31] DeLuca Stephanie, Duncan Greg J., Keels Micere, and Mendenhall Ruby. 2012. “The Notable and the Null: Using Mixed Methods to Understand the Diverse Impacts of Residential Mobility Programs.” In Neighbourhood Effects Research: New Perspectives, edited by van Ham Maarten, Manley David, Bailey Nick, Simpson Ludj, and Maclennan Duncan, 195–223. Dordrecht: Springer.

[R32] Desmond Matthew. 2016. Evicted: Poverty and Profit in the American City. New York: Penguin.

[R33] Doepke Matthias, Sorrenti Guiseppe, and Zilibotti Fabrizio. 2019. “The Economics of Parenting.” Annual Review of Economics 11: 55–84.

[R34] Duflo Esther. 2003. “Grandmothers and Granddaughters: Old-Age Pensions and Intrahousehold Allocation in South Africa.” World Bank Economic Review 17 (1): 1–25.

[R35] Duncan Greg J., Morris Pamela A., and Rodrigues Chris. 2011. “Does Money Really Matter? Estimating Impacts of Family Income on Young Children’s Achievement with Data from Random-Assignment Experiments.” Developmental Psychology 47 (5): 1263–79. 21688900 10.1037/a0023875 PMC3208322

[R36] Edin Kathryn J., and Shaefer H. Luke. 2015. $2.00 a day: Living on Almost Nothing in America. Boston: Houghton Mifflin.

[R37] Evans David K., and Popova Anna. 2017. “Cash Transfers and Temptation Goods.” Economic Development and Cultural Change 65 (2): 189–221.

[R38] Francesconi Marco, and Heckman James J.. 2016. “Child Development and Parental Investment: Introduction.” The Economic Journal 126 (596): 1–27.

[R39] Gaitz Jason, and Schurer Stefanie. 2017. “Bonus Skills: Examining the Effect of an Unconditional Cash Transfer on Child Human Capital Formation.” IZA Institute of Labor Economics Discussion Paper 10525.

[R40] Gennetian Lisa A., Conwell Jordan, and Daniels Becca. 2021. “How Do Low-Income Families Spend Their Money?” EconoFact, November 15. https://econofact.org/how-do-low-income-families-spend-their-money.

[R41] Gennetian Lisa A., Sarah Halpern-Meekin Lauren Meyer, Fox Nathan, Magnuson Katherine, Noble Kimberly G., and Yoshikawa Hirokazu. Forthcoming. “Cash to U.S. families at Scale: Behavioral Insights on Implementation from the Baby’s First Years Study.” In Using Cash Transfers to Build an Inclusive Society: A Behaviorally Informed Approach, edited by Soman D., Zhao J., and Datta S.. Toronto, ON: University of Toronto Press.

[R42] Gennetian Lisa A., and Shafir Eldar. 2015. “The Persistence of Poverty in the Context of Financial Instability: A Behavioral Perspective.” Journal of Policy Analysis and Management 34 (4): 904–36.

[R43] Gibson-Davis Christina, Keister Lisa A., and Gennetian Lisa A.. 2021. “Net Worth Poverty in Child Households by Race and Ethnicity, 1989–2019.” Journal of Marriage and Family 83 (3): 667–82. 34887593 10.1111/jomf.12742 PMC8653971

[R44] Halpern-Meekin Sarah, Edin Kathryn, Tech Laura, and Sykes Jennifer. 2015. It’s Not Like I’m Poor: How Working Families Make Ends Meet in a Post-welfare World. Oakland: University of California Press.

[R45] Ham John, and Shore-Sheppard Lara. 2005. “The Effect of Medicaid Expansions for Low-Income Children on Medicaid Participation and Private Insurance Coverage: Evidence from the SIPP”,Journal of Public Economics 89, 57–83

[R46] Hammond Samuel, and Xu Audrey. 2021. “Lead Us Not into Temptation: How the Child Tax Credit Creates Healthier Families.” Niskanen Center, July 15. https://www.niskanencenter.org/the-child-tax-credit-reduces-spending-on-alcohol-and-tobacco/.

[R47] Hardaway Cecily R., Sterrett-Hong Emma M., De Genna Natacha M., and Cornelius Marie D.. 2020. “The Role of Cognitive Stimulation in the Home and Maternal Responses to Low Grades in Low-Income African American Adolescents’ Academic Achievement.” Journal of Youth and Adolescence 49 (5): 1043–56. 32253658 10.1007/s10964-020-01217-x PMC7182545

[R48] Hastings Justine, Kessler Ryan and Shapiro Jesse M., 2021. “The Effect of SNAP on the Composition of Purchased Foods: Evidence and Implications,” American Economic Journal: Economic Policy, 13(3), pages 277–315.

[R49] Haushofer Johannes, and Fehr Ernst. 2014. “On the Psychology of Poverty.” Science 344 (6186): 862–67. 24855262 10.1126/science.1232491

[R50] Heckman James J. 2007. “The Economics, Technology and Neuroscience of Human Capability Formation.” Proceedings of the National Academy of Sciences 104 (3): 13250–55. 10.1073/pnas.0701362104 PMC1948899 17686985

[R51] Heckman James J., and Mosso Stefano. 2014. “The Economics of Human and Social Mobility.” Annual Review of Economics 6: 689–733. 10.1146/annurev-economics-080213-040753 PMC4204337 25346785

[R52] Heflin Colleen M. 2017. “The Role of Social Positioning in Observed Patterns of Material Hardship: New Evidence from the 2008 Survey of Income and Program Participation.” Social Problems 64 (4): 513–31.

[R53] Heflin Colleen, Sandberg John, and Rafail Patrick. 2009. “The Structure of Material Hardship in U.S. Households: An Examination of the Coherence behind Common Measures of Well-Being.” Social Problems 56 (4): 746–64.

[R54] Hill Heather D., Morris Pamela, Gennetian Lisa A., Wolf Sharon, and Tubbs Carly. 2013. “The Consequences of Income Instability for Children’s Well-Being.” Child Development Perspectives 7 (2): 85–90. 33042213 10.1111/cdep.12018 PMC7546433

[R55] Hoynes Hilary, Miller Doug, and Simon David. 2015. “Income, the Earned Income Tax Credit, and Infant Health.” American Economic Journal: Economic Policy 7 (1): 172–211.

[R56] Hoynes Hilary, Schanzenbach Diane Whitmore, and Almond Douglas. 2016. “Long-Run Impacts of Childhood Access to the Safety Net.” American Economic Review 106 (4): 903–34.

[R57] Iceland John, and Bauman Kurt J.. 2007. “Income Poverty and Material Hardship: How Strong Is the Association?” The Journal of Socio-Economics 36 (3): 376–96.

[R58] Iceland John, Kovach Claire, and Creamer John. 2021. “Poverty and the Incidence of Material Hardship, Revisited.” Social Science Quarterly 102 (1): 585–617. 10.1111/ssqu.12922 PMC11529799 39494105

[R59] Jaroszewicz Ania, Jachimowicz Jon, Hauser Oliver, and Jamison Julian. 2022. “How Effective Is (More) Money? Randomizing Unconditional Cash Transfer Amounts in the US.” July 5, 2022. https://papers.ssrn.com/sol3/papers.cfm?abstract_id=4154000.

[R60] Jones Damon, and Marinescu Ioana. Forthcoming. “The Labor Market Impacts of Universal and Permanent Cash Transfers: Evidence from the Alaska Permanent Fund.” American Economic Review: Economic Policy.

[R61] Jones Lauren E., Milligan Kevin, and Stabile Mark. 2019. “Child Cash Benefits and Family Expenditures: Evidence from the National Child Benefit.” Canadian Journal of Economics 52 (4): 1433–63.

[R62] Ariel Kalil, Ryan Rebecca and Corey Michael. 2012. Diverging Destinies: Maternal Education and the Developmental Gradient in Time with Children. Demography. 49 (4): 1361–83. 22886758 10.1007/s13524-012-0129-5 PMC4894844

[R63] Karpyn Allison E., Riser Danielle, Tracy Tara, Wang Rui, and Shen Ye. 2019. “The Changing Landscape of Food Deserts.” UNSCN Nutrition 44: 46–53. 32550654 PMC7299236

[R64] Knox V., Miller C., and Gennetian L. A.. 2000. Reforming Welfare and Rewarding Work: A Summary of the Final Report on the Minnesota Family Investment Program. New York: MDRC.

[R65] Kooreman Peter. 2000. “The Labeling Effect of a Child Benefit System.” American Economic Review 90 (3): 571–83.

[R66] Kornrich Sabino, and Furstenberg Frank. 2013. “Investing in Children: Changes in Parental Spending on Children, 1972–2007.” Demography 50: 1–23. 22987208 10.1007/s13524-012-0146-4

[R67] Knudsen Eric I., Heckman James J., Cameron Judy L., and Shonkoff Jack P.. 2006. “Economic, Neurobiological, and Behavioral Perspectives on Building America’s Future Workforce.” Proceedings of the National Academy of Sciences 103 (27): 10155–62. 10.1073/pnas.0600888103 PMC1502427 16801553

[R68] Levine R. A., Watts H., Hollister Robinson G., Williams W., O’Connor A., and Wilderquist K.. (2005). “A Retrospective On The Negative Income Tax Experiments: Looking Back At The Most Innovative Field Studies In Social Policy”. The Ethics And Economics Of The Basic Income Guarantee. 95–106

[R69] Ludwig Jens, Duncan Greg J., Gennetian Lisa A., Katz Lawrence F., Kessler Ronald C., Kling Jeffrey R., and Sanbonmatsu Lisa. 2013. “Long-Term Neighborhood Effects on Low-Income Families: Evidence from Moving to Opportunity.” American Economic Review 103 (3): 226–31.

[R70] Lugo-Gil Julieta, and Yoshikawa Hirokazu. 2006. “Assessing Expenditures on Children in Low-Income, Ethnically Diverse and Immigrant Families.” University of Michigan National Poverty Center Working Paper No. 06–36.

[R71] Lundberg Shelly. 2008. “Gender and Household Decision-Making.” In Frontiers in the Economics of Gender, edited by Bettio Francesca and Verashchagina Alina, 116–34. London: Routledge.

[R72] Mani Anandi, Mullainathan Sendhil, Shafir Eldar, and Zhao Jiaying. 2013. “Poverty Impedes Cognitive Function.” Science 341 (6149): 976–80. 23990553 10.1126/science.1238041

[R73] Mayer Susan E., and Jencks Christopher. 1989. “Poverty and the Distribution of Material Hardship.” The Journal of Human Resources 24 (1): 88–114.

[R74] Mayer Susan E., Kalil Ariel, Oreopoulos Philip, and Gallegos Sebastian. 2018. “Using Behavioral Insights to Increase Parental Engagement: The Parents and Children Together Intervention.” Journal of Human Resources. Published ahead of print, April 5. 10.3368/jhr.54.4.0617.8835R.

[R75] Meyer Bruce D., and Sullivan James X.. 2019. “Annual Report on U.S. Consumption Poverty: 2018.” https://leo.nd.edu/assets/339909/2018_consumption_poverty_report_1_.pdf. Accessed December 17, 2021.

[R76] Milligan Kevin, and Stabile Mark. 2011. “Do Child Tax Benefits Affect the Well-Being of Children? Evidence from Canadian Child Benefit Expansions.” American Economic Journal: Economic Policy 3 (3): 175–205.

[R77] Morduch Jonathan, and Schneider Rachel. 2017. The Financial Diaries: How American Families Cope in a World of Uncertainty. Princeton, NJ: Princeton University Press.

[R78] Mullainathan Sendhil, and Shafir Eldar. 2013. Scarcity: Why Having Too Little Means So Much. New York: Picador.

[R79] Mullins Joseph. 2019. “Designing Cash Transfers in the Presence of Children’s Human Capital Formation.” https://www.josephlyonmullins.com/CashTransfersChildren2019.pdf. Accessed December 17, 2021.

[R80] National Academies of Sciences, Engineering, and Medicine. 2019. “Consequences of Child Poverty.” In A Roadmap to Reducing Child Poverty, 67–96. Washington, DC: The National Academies Press. 31593382

[R81] Noble Claire, Sala Giovanni, Peter Michelle, Lingwood Jamie, Rowland Caroline, Gobet Fernand, and Pine Julian. 2019. “The Impact of Shared Book Reading on Children’s Language Skills: A Meta-analysis.” Educational Research Review 28: 100290.

[R82] Noble Kimberly G., Magnuson Katherine, Gennetian Lisa A., Duncan Greg J., Yoshikawa Hirokazu, Fox Nathan A., and Halpern-Meekin Sarah. 2021. “Baby’s First Years: Design of a Randomized Controlled Trial of Poverty Reduction in the United States.” Pediatrics 148 (4): e2020049702. 34475270 10.1542/peds.2020-049702 PMC8487960

[R83] Pilauskas Natasha, Jacobs Brian, Rhodes Elizabeth, Richard Kathrine and Shaefer H. Luke 2022. The COVID Cash Transfer Study: The Impacts of an Unconditional Cash Transfer on the Wellbeing of Low-Income Families. Working Paper. Gerald R. Ford School of Public Policy, University of Michigan.

[R84] Robinson Carly D., Lee Monica G., Dearing Eric, and Rogers Todd. 2018. “Reducing Student Absenteeism in the Early Grades by Targeting Parental Beliefs.” American Educational Research Journal 55 (6): 1163–92.

[R85] Rojas Natalia M., Yoshikawa Hirokazu, Gennetian Lisa A., Mayra Lemus Rangel Samantha Melvin, Noble Kimberly, Duncan Greg, and Magnuson Katherine. 2020. “Exploring the Experiences and Dynamics of an Unconditional Cash Transfer for Low-Income Mothers: A Mixed-Methods Study.” Journal of Children and Poverty 26 (1): 64–84.

[R86] Romich Jennifer L., and Weisner Thomas. 2000. “How Families View and Use the EITC: Advance Payment versus Lump Sum Delivery.” National Tax Journal 53 (4, pt. 2): 1245–65.

[R87] Rosenblatt Peter, and DeLuca Stephanie. 2012. “‘We Don’t Live Outside, We Live in Here’: Neighborhood and Residential Mobility Decisions among Low-Income Families.” City & Community 11 (3): 254–84.

[R88] Schazenbach Diane and Strain Michael. 2021. 2021. Employment Effects of the Earned Income Tax Credit: Taking the Long view. Tax Policy and The Economy. University of Chicago Press.

[R89] Schofield Heather, and Venkataramani Atheendar S.. 2021. “Poverty-Related Bandwidth Constraints Reduce the Value of Consumption.” Proceedings of the National Academy of Sciences 118 (35): e2102794118. 10.1073/pnas.2102794118 PMC8536330 34446552

[R90] Shah Anuj K., Mullainathan Sendhil, and Shafir Eldar. 2012. “Some Consequences of Having Too Little.” Science 338 (6107): 682–85. 23118192 10.1126/science.1222426

[R91] Short Kathleen S. 2005. “Material and Financial Hardship and Income-based Poverty Measures in the USA.” Journal of Social Policy 34 (1): 21–38.

[R92] Stanczyk Alexandra B. 2020. “The Dynamics of Household Economic Circumstances Around a Birth.” Demography 57 (1): 1271–96. 32705567 10.1007/s13524-020-00897-1

[R93] Troller-Renfree Sonya, Costanzo Molly A., Duncan Greg J., Magnuson Katherine, Gennetian Lisa A., Yoshikawa Hirokazu, Sarah Halpern-Meekin Nathan Fox, Noble Kimberly G.. 2022. “The Impact of Poverty Reduction Intervention on Infant Brain Activity.” Proceedings of the National Academy of Sciences 119 (5): e2115649119. 10.1073/pnas.2115649119 PMC8812534 35074878

[R94] USDA Economic Research Service. 2021. “Food Security and Nutrition Assistance.” https://www.ers.usda.gov/data-products/ag-and-food-statistics-charting-the-essentials/food-security-and-nutrition-assistance/. Accessed December 14, 2021.

[R95] U.S. Department of Health and Human Services, Administration for Children and Families. n.d. “State Disconnection Policies: Louisiana.” https://liheapch.acf.hhs.gov/Disconnect/disconnect.htm#la. Accessed December 22, 2021.

[R96] Wade Sally M. 2004. “Parenting Influences on Intellectual Development and Educational Achievement.” Handbook of Parenting: Theory and Research for Practice, edited by Hoghughi Masud and Long Nicholas, 198–212. London: Sage Publications.

[R97] Westfall Peter H., and Young S. Stanley. 1993. Resampling-Based Multiple Testing: Examples and Methods for p-Value Adjustment. New York: Wiley.

[R98] Winship Scott. 2021. Reforming Tax Credits to Promote Child Opportunity and Aid Working Families. American Enterprise Institute. https://www.aei.org/research-products/report/reforming-tax-credits-to-promote-child-opportunity-and-aid-working-families/.

[R99] Yoo Paul Y. Greg J. Duncan, Magnuson Katherine, Fox Nathan A., Sarah Halpern-Meekin Hirokazu Yoshikawa, and Noble Kimberly G.. 2022. “Unconditional Cash Transfers and Maternal Substance Use: Findings from a Randomized Control Trial of Low-Income Mothers with Infants in the U.S.” BMC Public Health 22 (1): 897. 35513842 10.1186/s12889-022-12989-1 PMC9070980

[R100] Zelizer Viviana A. 2011. “Part Two: The Social Meaning of Money.” In Economic Lives: How Culture Shapes the Economy. Princeton, NJ: Princeton University Press

